# Regional feature purification contrastive learning for wheat biotic stress detection

**DOI:** 10.3389/fpls.2025.1523214

**Published:** 2025-10-14

**Authors:** Junming Chen, Yu-Xuan Chen, Sheng-He Xu

**Affiliations:** ^1^ University of Petroleum (East China), Qingdao, China; ^2^ School of Optoelectronic Engineering, Changchun University of Science and Technology, Changchun, China; ^3^ School of Mathematics and Statistics, Fuyang Normal University, Fuyang, China

**Keywords:** wheat diseases classification, image classification, contrastive learning, deep learning, wheat biotic stress detection

## Abstract

The identification of wheat infections has always been a considerable problem in agricultural forecasting. This paper presents an automated classification framework for wheat illnesses utilising region feature purification contrastive learning, which combines unsupervised representation learning with label mutual information maximisation to improve feature extraction and classification efficacy. The integration of the W-Paste approach enhances the model’s resilience to input perturbations, hence augmenting its out-of-distribution detection efficacy. Additionally, the creation of a feature purification encoder enhances feature consistency by reducing interference via reverse learning, resulting in a significant improvement in classification accuracy. Attaining an average classification accuracy of 98.01% on public datasets illustrates the remarkable performance, efficacy, and resilience of our system in intricate situations. This study presents a novel and pragmatic approach for the automated identification of wheat illnesses, laying a robust groundwork for the progression of intelligent agriculture. The ongoing enhancement of the suggested framework is anticipated to advance the early detection and accurate diagnosis of wheat illnesses, hence promoting more effective crop management and sustainable agricultural development.

## 1 Introduction

Agriculture is fundamental to society, supporting the livelihoods of billions globally. With the continuous increase in the world population, guaranteeing a stable and secure food supply has become increasingly essential. Apart from supplying food, agriculture is crucial for economic development and creating numerous employment possibilities, positively impacting millions worldwide. Wheat, as a crucial cereal crop, is fundamentally connected to human life and advancement. It ranks among the most widely consumed crops worldwide, with data showing that the yearly per capita consumption surpasses 50 kilogrammes in 102 nations.

The increasing population is driving a consistent rise in the need for wheat production. Wheat plants are particularly vulnerable to numerous diseases, representing a significant risk to global production. Annually, substantial crop losses occur owing to viral and bacterial diseases, with wheat-producing nations experiencing potential yield declines between 45% and 100%. Common wheat diseases, including rust (Prasad et al., 2020), powdery mildew (Wang et al., 2023), smut (Putterill, 1920), and fusarium head blight (Dweba et al., 2017), represent serious risks to farming, markedly reducing both quality and production while causing considerable economic harm. Therefore, to reduce output losses and preserve crop health, proactive protective strategies, such as early diagnosis and intervention, are essential.

Conventional disease detection in wheat depends on manual field assessments. This strategy has become progressively difficult as it requires considerable time and effort from farmers and pathologists to precisely evaluate the degree of infection. Manual detection is laborious, expensive, resource-demanding, and necessitates specialised skill, frequently resulting in delayed reactions during extensive epidemics. In response to these obstacles, researchers and farmers are diligently investigating accurate, swift, automated, and economical disease detection techniques.

In recent years, breakthroughs in artificial intelligence have established deep learning as a transformative instrument for agricultural disease identification, producing exceptional outcomes. Deep learning techniques have attained significant accuracy at comparatively modest expenses, providing innovative solutions for early disease identification in agriculture. This technique improves detection efficiency and provides farmers with scientific and dependable management tools, therefore protecting wheat output and fostering sustainable agriculture development.

Jiang et al (2022) assessed seven classical convolutional neural networks (VGG-16, Inception-v3, ResNet-50, DenseNet-121, EfficientNet-B6, ShuffleNet-v2, and MobileNetV3), analysing the efficacy of various training strategies in detecting wheat leaf diseases, including powdery mildew, leaf rust, and stripe rust. The Inception-v3 model attained a peak recognition accuracy of 92.5% on the test dataset.

Li et al (2023b) introduced an effective deep learning model, PMVT, derived from MobileViT for the real-time diagnosis of plant diseases. The model was evaluated on various datasets, including wheat, coffee, and rice, attaining maximum recognition rates of 93.6%, 85.4%, and 93.1%, respectively.

Alharbi et al (2023) proposed a few-shot learning model based on EfficientNet, including attention techniques to improve feature selection. Their methodology attained a classification accuracy of 93.19%, successfully recognising 18 different wheat illnesses.

Cheng et al (2023) created a location-aware detection model that incorporates a positional attention module to extract spatial information from feature maps and produce attention maps, thus enhancing the identification of diseased areas. This module was integrated into architectures including AlexNet, VGG, MobileNet, ResNet, and GoogLeNet, with ResNet achieving the highest performance, attaining an accuracy of 96.4% in testing.

Fang et al (2023) combined residual modules with Inception modules to create a lightweight multi-scale architecture called Inception-ResNet-CE (IRCE). The model integrated CBAM and ECA attention modules into the residual blocks to improve the extraction of disease-related characteristics and reduce interference from intricate backgrounds. It attained accuracy ratings of 99.74%, 96.7%, and 96.7% on the Plant-Village, CGIAR, and Wheat Leaf datasets, respectively.

Nigus et al (2024 introduced SRNet for the diagnosis of wheat stem rust. The research workflow included picture preprocessing, segmentation, feature extraction, and softmax classification. In the segmentation phase, an adaptive threshold method was utilised to identify sick areas, while Gabor filters were implemented to augment textural characteristics. SRNet attained a test accuracy of 92.01% on a proprietary dataset.

Li et al (2023) created the GhostNetV2 model to address wheat stripe rust. The model improved inter-channel communication in Ghost modules by rearranging channels and substituted five G-bneck layers with Fused-MBConv blocks to expedite training. Furthermore, the SE attention mechanism was replaced with ECA to enhance recognition performance. GhostNetV2 attained an accuracy of 95.44% on the Yellow-Rust-19 dataset. Jiang et al (2021) enhanced the VGG16 model by multitask learning, utilising transfer learning and alternating learning procedures with pretrained ImageNet models. Experimental findings indicated that the multi-task methodology surpassed single-task models, reusable models, ResNet50, and DenseNet121, attaining recognition accuracies of 97.22% for rice leaf illnesses and 98.75% for wheat leaf diseases.

Bao et al (2021b) introduced a metric learning approach utilising the Elliptical Maximum Margin Criterion (E-MMC) to ascertain the type and severity of stripe rust and powdery mildew infections. This method utilised the Otsu algorithm for lesion segmentation and applied gradient ascent to enhance the metric matrix, hence minimising feature redundancy. The approach attained a maximum recognition accuracy of 94.16%.

Genaev et al (2021) proposed a technique for mitigating data deterioration utilising image hashing algorithms and employed the EfficientNet network for illness identification. The network attained an accuracy of 94.2% in identifying several fungal wheat illnesses, such as leaf rust, stem rust, and powdery mildew, through the application of data augmentation and picture style transfer techniques.

Bao et al (2021a) created a lightweight model, SimpleNet, for the automatic detection of wheat ear disorders from natural field photos. SimpleNet incorporated the CBAM module, which amalgamates spatial and channel attention methods, to augment feature representation for disease diagnosis. The model attained an accuracy of 94.1% on the test dataset.

Pandey (Pandey and Jain, 2022) proposed an Attention-Dense Learning (ADL) mechanism that combines hybrid S-shaped attention learning with the dense learning process of convolutional neural networks (CNNs). Experimental findings on the PlantVillage dataset indicated that this framework attained an accuracy of 96.57%.

Pan et al (2022) introduced a rust detection model, WREL, utilising ensemble learning that integrates networks like ResNet152, VGG, ResNet101, and DenseNet201. The experiments demonstrated that the model accurately forecasts rust infections and reduces agricultural losses.

Hayit et al (2021) created the Yellow-Rust-Xception model for the detection of stripe rust in wheat leaves and the assessment of its severity. The model attained 91% accuracy on the test dataset.

Despite the considerable promise of current methodologies, numerous constraints persist in tackling the intricacies and variability of wheat leaf disease and pest imagery. The efficacy of numerous contemporary methods is significantly contingent upon the quality, diversity, and representativeness of the training datasets, which undermines their ability to forecast unfamiliar illness patterns. Moreover, the implementation of ensemble models considerably elevates system complexity and computing duration. Numerous techniques depend on the extraction of image-level semantic characteristics, considering background information as contextual, which may occasionally capture distinguishing cues. Nonetheless, actual wheat leaf photos frequently suffer from intricate backdrops, unfavourable weather conditions, focus blur, occlusions, and extraneous objects (Li et al., 2023a). These factors can significantly diminish image quality, resulting in minimal inter-class variances and substantial intra-class changes among disorders. Furthermore, samples within the same class may have significantly divergent environmental contexts, whilst samples from distinct subclasses may possess remarkably like environments, so rendering background information inaccurate and a possible source of confounding in disease categorisation.

Attention mechanisms, when employed to activate discriminative features, frequently misidentify ambiguous peripheral information, resulting in inaccurate localisation of regions pertinent to wheat leaf disease classification. To tackle these challenges, it is essential to develop a model that comprehensively investigates the intrinsic features of different wheat leaf diseases, emphasising local regions that significantly aid in classification. Ensuring strong feature consistency while minimising reliance on background information is essential for improving the model’s robustness in complex heterogeneous images. This necessitates the implementation of more sophisticated feature selection methods to accurately identify the most pertinent features for each class, thereby reducing the impact of noise on detailed disease classification. Our research aims to establish robust feature consistency from sparsely distributed data to facilitate the automated recognition of various wheat leaf diseases. This method enhances classification accuracy and offers more dependable disease management strategies for wheat production.

Self-supervised contrastive learning creates strong feature uniformity in the embedding space by grouping positive samples with anchors and separating negative samples. This method has shown significant effectiveness in natural image domains. However, the structural and distributional similarities among wheat leaf disease samples often result in high similarity among anchors from different categories, which can impede the effectiveness of self-supervised contrastive learning. Supervised contrastive learning addresses this limitation by incorporating label information, thereby mitigating the challenge of anchor similarity. This approach proves to be particularly effective in scenarios with limited labelled data.

The primary benefit of supervised contrastive learning is its ability to diminish reliance on extensive labelled datasets by utilising the similarities among various but related samples to develop robust representations. This strategy promotes accelerated learning and enhanced accuracy in inference. This study introduces the integration of enhanced supervised contrastive learning with a feature purification encoder for the automatic detection of wheat leaf diseases. We maximise mutual information among various data through unsupervised representation learning for feature extraction, while also incorporating label-based mutual information maximisation for image classification. This method introduces supplementary regional constraints to self-supervised contrastive learning by deriving positive instances from samples belonging to the same class as the anchor, instead of depending exclusively on data augmentation of the anchor, which is the conventional practice in self-supervised learning. This strategy seeks to optimise inter-class separation and minimise intra-class distances among wheat leaf disease samples, thus decreasing the likelihood of misclassification in difficult cases and improving classification accuracy.

To augment the semantic diversity of wheat leaf disease representations, we refine supervised contrastive learning to provide robust feature consistency, hence enhancing the model’s efficacy in out-of-distribution detection. To achieve this objective, we devised the W-Paste approach to produce positive cases. W-Paste emulates authentic scenarios related to several kinds of wheat leaf diseases, hence augmenting the model’s resilience to input deterioration and strengthening its capacity to detect out-of-distribution instances. To investigate appropriate semantic representations of wheat leaf diseases, we developed a feature purification encoder. These unique techniques enhance classification performance and offer strong support for accurate diagnosis of wheat leaf diseases.

## 2 Method

**Figure 1:**
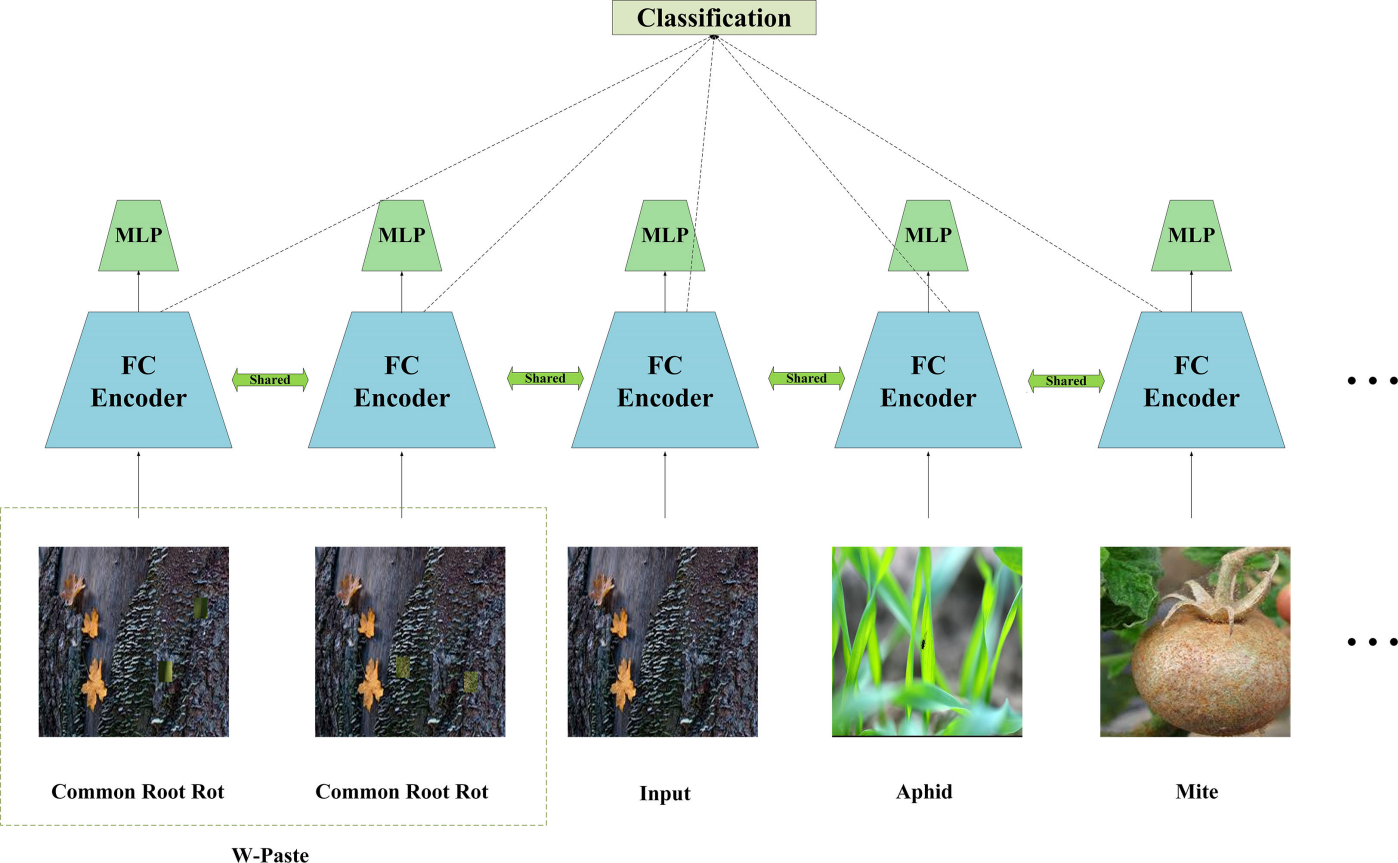
An overview of our proposed regional feature purification contrastive learning.

This section presents a detailed elucidation of the suggested two-stage architecture for automated wheat disease diagnosis. We initially employ the W-Paste technique twice on the input data to generate two unique views from the same batch, thus creating positive samples. This approach emulates real-world samples and introduces little semantic variations, improving the network’s generalisation ability across new data distributions. We subsequently produce 2048-dimensional normalised embeddings via a feature purification encoder and transmit them through a projection network, which is eliminated throughout the testing step. The supervised contrastive loss is calculated using the outputs of the projection network. To do post-training classification, we immobilise the encoder and train a linear classifier atop it. **Figure 1** presents a visual representation of this process.

### 2.1 W-Paste

In practical situations, environmental variables make field photographs of wheat exceedingly intricate. Furthermore, the diminutive size of many disease symptoms and the resemblance across various disease appearances further hinder recognition accuracy. Moreover, photos of diseases frequently have low contrast and cluttered backgrounds, complicating feature extraction. While supervised contrastive learning can group samples of the same class with analogous characteristics in the feature space, significant intra-class variances and inter-class similarities impede the formation of feature consistency.

**Figure 2:**
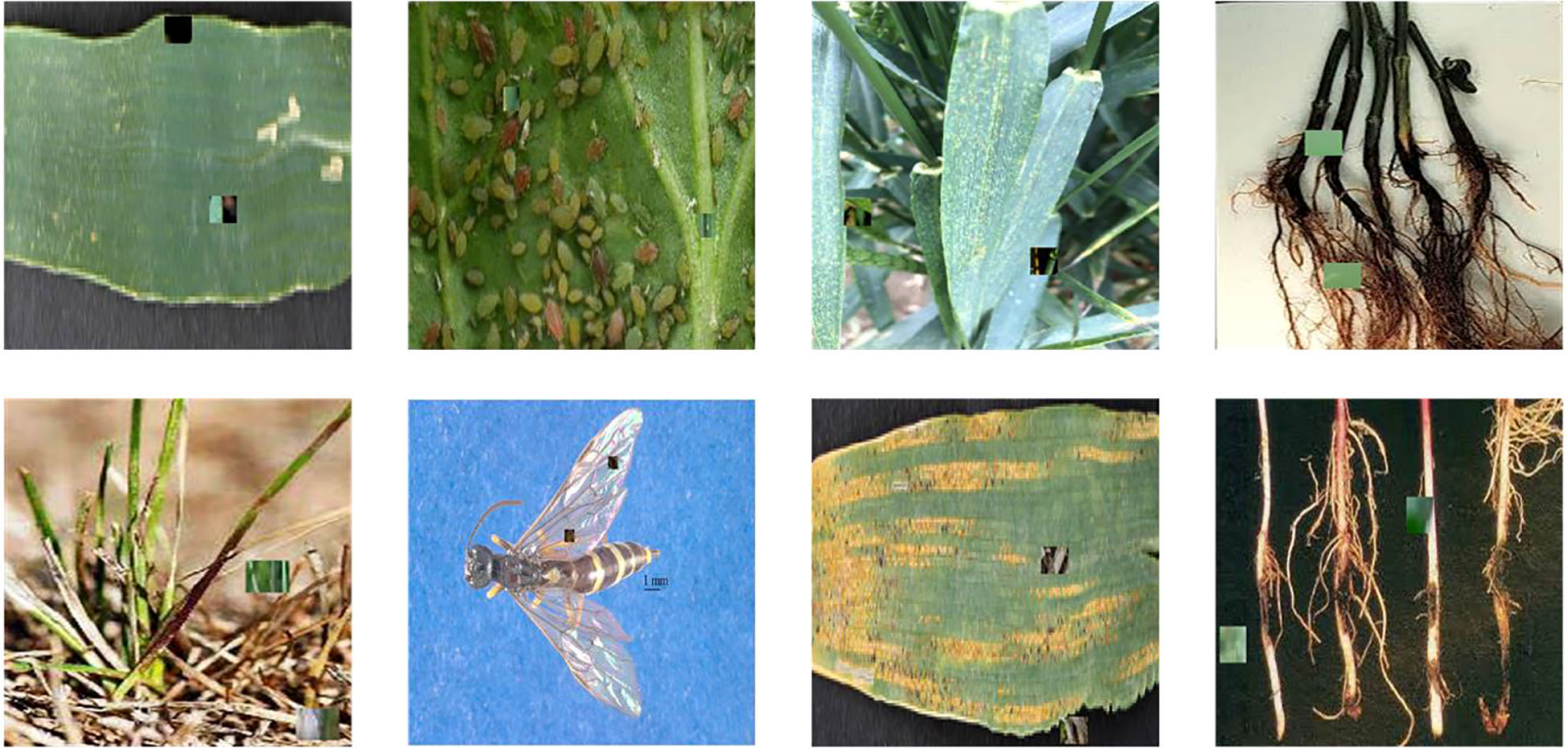
Visualization of W-paste.

To tackle these problems, we utilise the W-Paste technique to replicate intricate wheat leaf diseases in real-world scenarios for generating positive samples, thus incorporating out-of-distribution data points and augmenting the network’s ability to recognise out-of-distribution instances. We randomly eliminate two tiny rectangular portions from the samples, utilising 15×15 patches, and subsequently fill these areas with segments from other samples to provide varied semantic perturbations. **Figure 2** demonstrates that our methodology significantly enhances sample complexity. The produced samples closely mimic genuine wheat leaf diseases, hence improving the model’s generalisation capability. In this technique, two random augmentations are added to each input sample to create positive pairs, representing varied viewpoints of the same sample. Concurrently, random samples bearing identical labels in the queue exhibit comparable feature consistency, whereas the existence of disparate labels further restricts the model’s learning process. This varied distribution greatly enhances the practical effectiveness of contrastive learning.

### 2.2 Feature cleansing encoder

Neural network layers are known to have significant differences in feature learning abilities: lower-level features frequently contain considerable noise, whereas higher-level semantic features exhibit enhanced discriminative strength. Despite the impressive capability of CNNs in feature representation, its accuracy in localizing uncertain areas is still inadequate. The feature purification encoder’s architecture includes a structure consisting of five residual blocks to resolve this issue. The initial two residual blocks are allocated for low-level feature extraction; hence, we implement interference feature filtering modules at these phases to refine the features.

The Interference feature filtering module includes an attention module and a feature subtraction module. For the input feature X, the interference feature is first enhanced through attention module.

#### 2.2.1 Attention module

**Figure 3:**
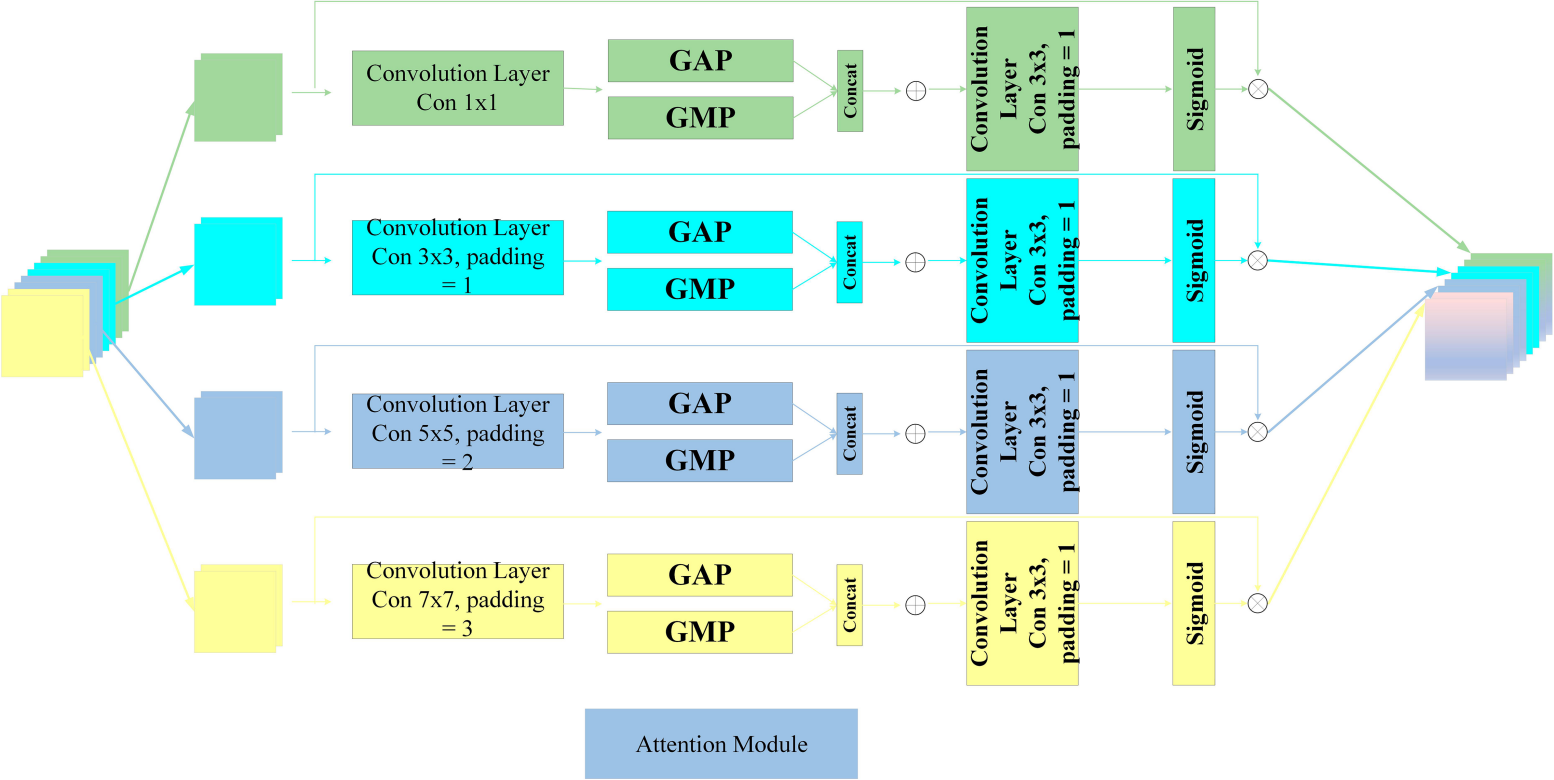
An overview of attention module.


The attention module’s architecture, seen in **Figure 3**, initiates by dividing the input feature *X* along the channel dimension into four subspaces. Convolutional kernels of dimensions 1 × 1, 3 × 3, 5 × 5, and 7 × 7 are utilized to extract features from each subspace. This segmentation technique facilitates the concurrent processing of input tensors at many scales, producing feature maps tailored to each kernel type. Each segment individually captures multi-scale spatial information, thereby establishing local cross-channel interactions. Subsequently, global average pooling and global max pooling procedures are executed on each set of feature maps, which are then concatenated along the channel dimension (As shown in **Equations 1**–**4**).

(1) $$F\left(x_{i}\right)=F_{3\times 3}\left(F^{\prime }\right)$$

(2) $$w=\sigma \left(F\left(x_{i}\right)\right)$$

(3) $$F_{i}=F\times w$$

(4) $$F\left(X_{i}\right)=con\left(F_{1},F_{2},F_{3},F_{4}\right)$$

#### 2.2.2 Feature subtraction module

The captured interference features are subtracted from the intermediate features (the third residual block and the fourth residual block) to remove irrelevant or confusing local information (As shown in **Equations 5**, **6**).


(5) $$G\left(X_{1}\right)=F\left(X_{3}\right)-F\left(X_{1}\right)$$

(6) $$G\left(X_{2}\right)=F\left(X_{4}\right)-F\left(X_{2}\right)$$

### 2.3 Projection network

We configured a multi-layer perceptron with 2,048 neurons in its hidden layer and an output vector dimension of 256. Following the self-supervised contrastive learning approach proposed by Tian et al (Tian et al., 2020), we adopted a similar technique in our study. Specifically, the network outputs are normalized onto a unit hypersphere, allowing the use of inner products to measure distances within the projection space. As with typical self-supervised contrastive training, the projection network is discarded after the contrastive training phase. Thus, the total parameter count in our test model remains unaffected.

### 2.4 Classification network

After finalising the initial phase of contrastive learning, we advance to the subsequent step by immobilising the encoder and developing a linear classifier to serve as the urinary sediment classification layer. We subsequently refine the wheat leaf disease classification model by training it for a mere 20 epochs.

### 2.5 Supervised contrastive losses

One may reconcile fully supervised learning (SL) with self-supervised learning (SSL) by employing SupCon to formulate the supervised contrastive learning loss function (Khosla et al., 2020). The primary objective of SupCon is to reduce the distance between positive samples, which are those that belong to the same class. A contrastive loss function is utilized to achieve this purpose, meticulously designed to consider both positive and negative pairings concurrently. The loss function encourages the model to enhance the distinction between different classes in the normalized feature space. This method enables the model to recognize shared traits across instances of the same category while simultaneously exhibiting remarkable skill in distinguishing differences among other categories. The SupCon loss function is articulated by the subsequent equation:


(7) $${\text{L}}^{\text{sup }}=\sum_{\text{i}\in \text{I}}\frac{-1}{\left|\text{P}\left(\text{i}\right)\right|}\sum_{\text{p}\in \text{P}\left(\text{i}\right)}\text{log }\frac{\exp\;\left(z_{\text{i}}\cdot z_{\text{p}}/\tau \right)}{\sum_{\text{a}\in \text{A}\left(\text{i}\right)}\exp\;\left(z_{\text{i}}\cdot z_{\text{a}}/\tau \right)}$$

Here, $P\left(i\right)\;\equiv \left\{p\;\in \;A\left(i\right)\;:\;{\tilde{y}}_{p}={\tilde{y}}_{i}\right\}$ is the set of indices of all positives in the multiviewed batch distinct from i, and |*P*(*i*)| is its cardinality. Loss have the following desirable properties: Supervised losses urge the encoder to give all entries from the same class tightly aligned representations, improving representation space clustering. More negatives increase contrast (Grill et al., 2020). Hard positive/negative mining ability from within. Using normalized representations, the loss in Equation 7 creates a gradient structure, resulting in implicit positive/negative mining. Hard positives/negatives (those against which continuing to contrast the anchor greatly benefits the encoder) have large gradient contributions, while easy ones have small ones. Hard positives have an asymptotically increasing effect as negatives increase.

## 3 Experiments and results

### 3.1 Dataset

Public Datasets 1:This research utilises the “Wheat Disease Detection Dataset” for the classification of wheat illnesses. The dataset, initially created by Safarijalal et al (Safarijalal et al., 2022), comprises three categories: Brown Rust, Healthy, and Yellow Rust. The Wheat Disease Detection Dataset is accessible to the public at the following URL: https://www.kaggle.com/sinadunk23/behzad-safari-jalal. Our trials utilised a total of 3,679 photos, with comprehensive details included in **Table 1**. The dataset was divided into training, validation, and test sets in a 7:2:1 ratio.

**Table 1:** Dataset details.

Public datasets 1	Number	Public datasets 2	Number
Brown Rust	1128	Aphid	6955
Healthy	1395	Black Rust	7540
Yellow Rust	1156	Blast	5063
		Brown Rust	5703
		Common Root Rot	1183
		Fusarium Head Blight	1581
		Healthy	1093
		Leaf Blight	1076
		Mildew	1237
		Mite	1208
		Septoria	832
		Smut	832
		Stem fly	832
		Tan spot	832
		Yellow Rust	832

Public Datasets 2:Furthermore, we employed the “Wheat Plant Diseases Dataset” supplied from the Kaggle platform, which comprises 14,154 photos of wheat leaves. **Table 1** offers a detailed account of the dataset. The dataset, available at https://www.kaggle.com/sinadunk23/behzad-safari-jalal, is divided into fifteen unique categories: Aphid, Black Rust, Blast, Brown Rust, Common Root Rot, Fusarium Head Blight, Healthy, Leaf Blight, Mildew, Mite, Septoria, Smut, Stem Fly, Tan Spot, and Yellow Rust. The data was divided into training, validation, and test sets in a 7:2:1 ratio.

### 3.2 Experimental settings

We conduct experiments on RTX3090 GPU. In the first stage of training, the batch size is set to 8, and we train for 200 epochs. At the same time, after 15 epochs, we replace the subtraction operation of the interference feature in the feature subtraction module with the addition operation. In the second stage of training, the cross entropy loss function is used to update network.

### 3.3 Experimental results

To validate the effectiveness of our proposed method in leveraging label information, we conducted a comparative analysis of classical convolutional networks, Transformer architectures, and unsupervised contrastive learning across two public datasets, with detailed results presented in **Table 2**. Supervised learning significantly enhanced model performance relative to unsupervised methods by employing label-guided learning, resulting in superior outcomes across several evaluation metrics. Our supervised contrastive learning approach shown substantial effectiveness in imposing stringent constraints, enabling a more precise characterization of wheat disease traits and significantly improving classification performance.

**Table 2:** Experimental results of different classification models.

Method	Dataset	Accuracy (%)	Precision (%)	Recall (%)	F1-score (%)
VGG19 (Simonyan and Zisserman, 2014)	Public Dataset 1	87.85	88.02	87.77	87.89
ConvNeXt (Liu et al., 2022)	Public Dataset 1	88.01	88.68	89.77	89.22
GoogLeNet (Szegedy et al., 2015)	Public Dataset 1	91.07	91.02	91.11	91.06
MobileNet (Howard, 2017)	Public Dataset 1	92.08	92.33	92.46	92.39
ResNet50 (He et al., 2016)	Public Dataset 1	93.21	92.92	93.16	93.04
DenseNet121 (Huang et al., 2017)	Public Dataset 1	93.65	94.74	94.02	94.38
Transformer (Vaswani, 2017)	Public Dataset 1	93.68	94.25	94.16	94.20
Swin Transformer (Liu et al., 2021)	Public Dataset 1	94.69	95.79	94.56	95.17
SimCLR (Chen et al., 2020)	Public Dataset 1	95.41	95.60	95.63	95.61
MoCo v3 (He et al., 2020)	Public Dataset 1	95.56	96.12	95.64	95.88
Ours	Public Dataset 1	98.19	98.24	98.37	98.22
VGG19 (Simonyan and Zisserman, 2014)	Public Dataset 2	86.77	87.43	86.44	86.93
ConvNeXt (Liu et al., 2022)	Public Dataset 2	87.80	87.72	87.40	87.56
GoogLeNet (Szegedy et al., 2015)	Public Dataset 2	87.83	87.40	88.16	87.78
MobileNet (Howard, 2017)	Public Dataset 2	89.08	89.37	89.37	89.36
ResNet50 (He et al., 2016)	Public Dataset 2	91.57	90.01	91.04	90.52
DenseNet121 (Huang et al., 2017)	Public Dataset 2	92.89	93.05	93.66	93.35
Transformer (Vaswani, 2017)	Public Dataset 2	92.66	93.48	93.31	93.39
SimCLR (Chen et al., 2020)	Public Dataset 2	93.59	95.22	94.06	94.64
Swin Transformer (Liu et al., 2021)	Private Dataset 2	94.48	95.87	95.49	95.68
MoCo v3 (He et al., 2020)	Public Dataset 2	95.86	94.81	96.09	95.45
Ours	Public Dataset 2	98.01	98.01	98.00	98.00

Experimental results revealed that convolutional networks had less local inductive capacity on datasets with greater class diversity, due to the absence of prominent features. Moreover, pooling processes diminished fine-grained information and overlooked the linkages between local and global contexts, hence limiting the networks’ ability to appropriately represent characteristics. The Swin-Transformer outperformed ResNet50 by more effectively encoding positional information and managing global dependencies.

Our methods retained critical information throughout the preliminary training phase by removing extraneous aspects that obstructed clarity. Unsupervised contrastive learning exhibited a considerable dependence on the quality and quantity of images and was especially sensitive to the creation of positive sample pairings. Our technique enhanced the creation of intra-class positive pairs, minimizing the distance between samples of the same class in the representation space, thereby facilitating accurate extraction and aggregation of critical characteristics. This enabled us to utilize label information more efficiently, even with limited data, to create substantial positive pairs and precisely represent complex image features in wheat disease classification.

We investigated the impact of several methods for generating positive sample pairs through image augmentation techniques in the context of supervised contrastive learning. **Table 3** demonstrates that generating positive couples by random image cropping and feature occlusion might result in localized anomalies, hence enhancing the model’s generalization capacity. The findings consistently demonstrate that our proposed W-Paste methodology outperforms other augmentation techniques. This strategy improves the model’s focus on specific wheat disease regions, hence increasing its capacity to identify out-of-distribution cases.

**Table 3:** Data augmentation.

Data augmentation	Accuracy (%)	Precision (%)	Recall (%)	F1-score (%)
Rotate	92.54	92.53	91.76	92.14
Cutout (DeVries, 2017)	94.35	94.47	93.69	94.08
Cutmix (Yun et al., 2019)	95.38	94.49	94.59	94.54
W-Paste	98.01	98.01	98.00	98.00

We assessed our proposed method against other state-of-the-art wheat disease classification techniques, with results presented in **Table 4**. Although several algorithms enhance feature extraction through attention mechanisms, these mechanisms often neglect the critical attributes of wheat diseases, hence limiting classification effectiveness. In contrast, our methodology emphasizes the early collection of interfering features, which is particularly crucial when dealing with real-world wheat images characterized by low contrast and complex backgrounds. By identifying and analyzing these redundant features, we effectively remove superfluous information in the early stages of feature extraction, ensuring the model focuses on the most salient low-level features. Moreover, the incorporation of label information reinforces geographic constraints within the feature space for each condition. Our solution, combined with the new W-Paste technique for creating positive sample pairs, significantly enhances the generalization capability of automated wheat disease diagnosis and guarantees robust feature consistency.

**Table 4:** Performances comparison with state-of-the-art method.

Model	Recall (%)	Specificity (%)	AP (%)	F1-score (%)
Pandey and Jain (2022)	89.75	93.56	87.95	91.62
Hayit et al (2021)	91.21	92.96	89.71	92.08
Li et al (2023)	94.41	94.18	93.67	93.92
Ours	98.01	98.01	98.00	98.00

**Figure 4:**
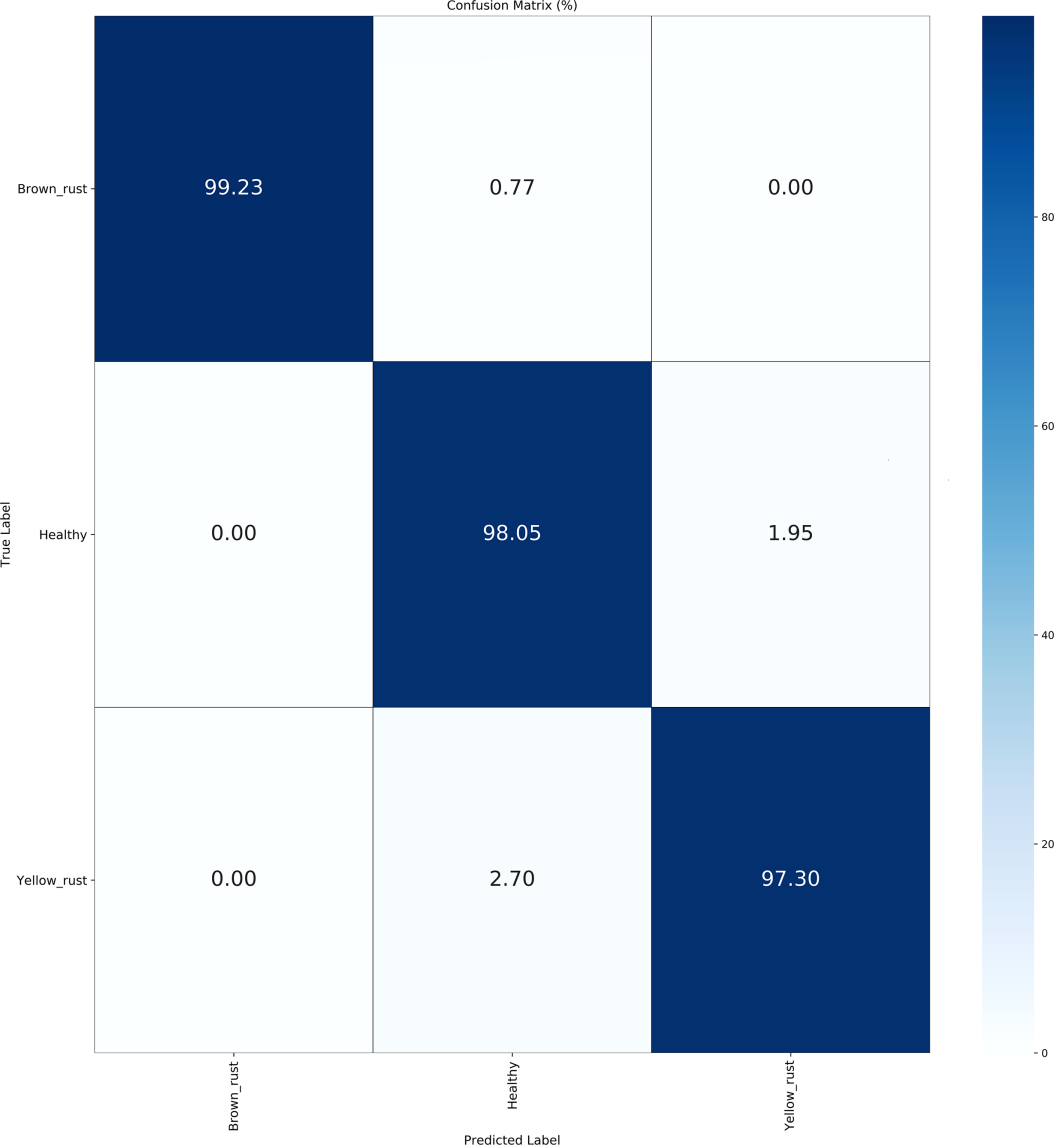
Confusion matrix for public datasets 1.

**Figure 5:**
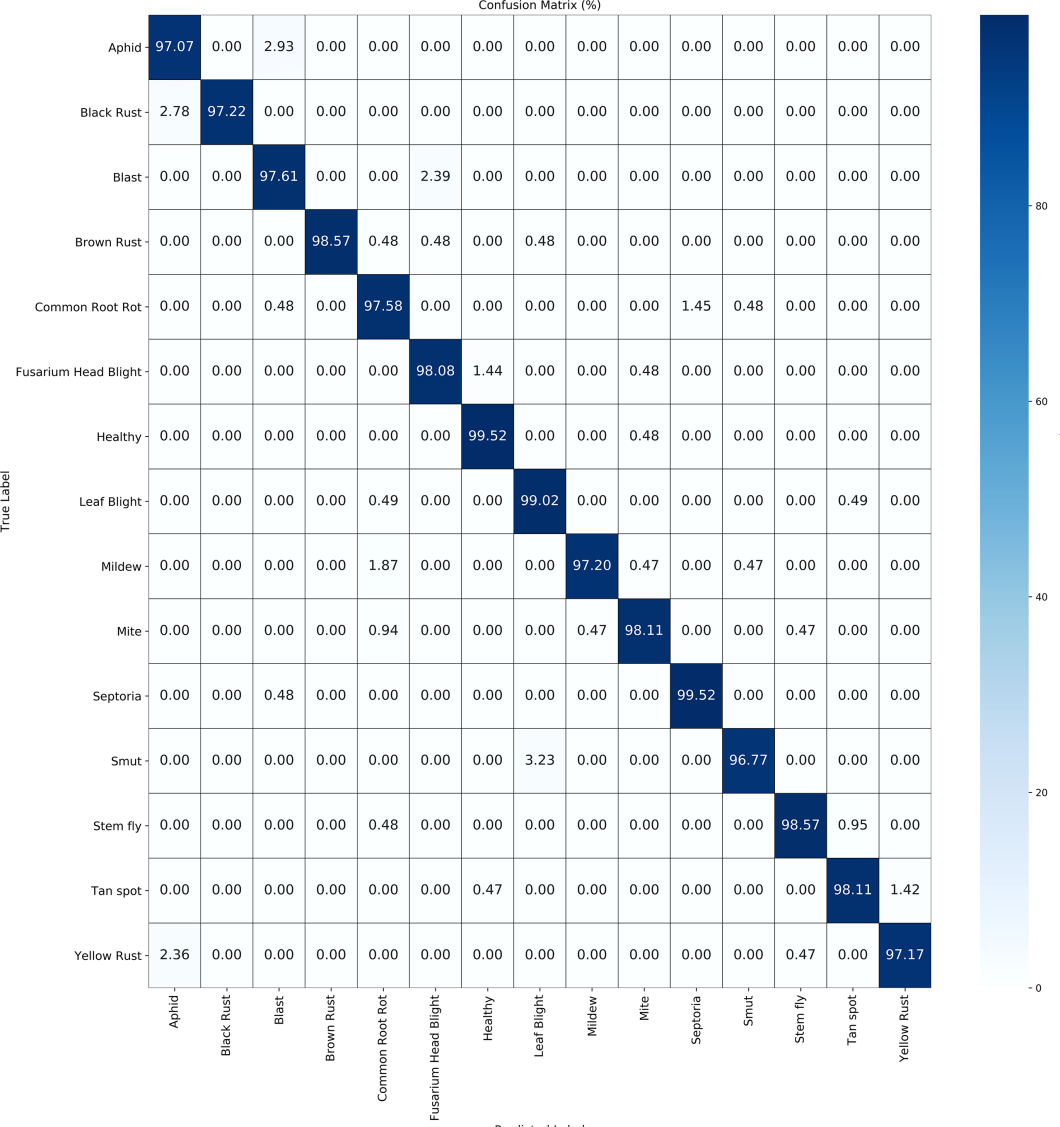
Confusion matrix for public datasets 2.

**Figure 6:**
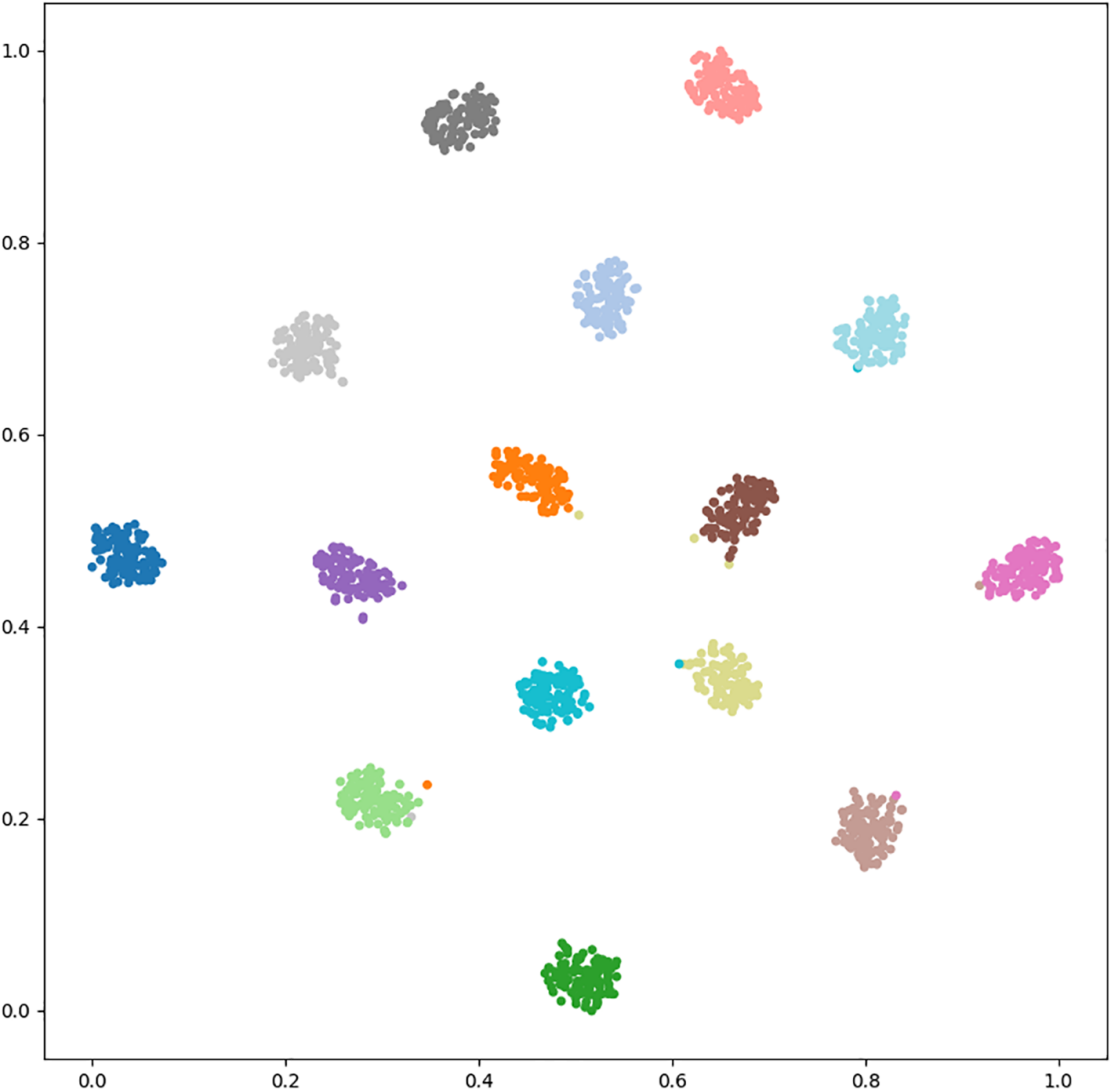
t-SNE visualization.

**Table 5** illustrate the detailed classification results of our methodology across 15 categories of wheat diseases in publically available datasets. **Figure 4** and **Figure 5** illustrate the confusion matrices for both datasets, providing improved insights into the classification effectiveness of our proposed technique. Additionally, we extracted features from the final layer and condensed them into two-dimensional vectors using t-SNE, as depicted in **Figure 6**. The visualization clearly demonstrates significant inter-class distances and tight intra-class distributions, highlighting the effectiveness of our approach in enhancing inter-class separation while minimizing intra-class volatility.

**Table 5:** Public dataset 2–15 classification results.

Class	Accuracy (%)	Precision (%)	Recall (%)	F1-score (%)
Aphid	97.07	94.76	97.07	95.90
Black Rust	97.22	100.00	97.22	98.59
Blast	97.61	96.22	97.60	96.91
Brown Rust	98.57	100.00	98.57	99.28
Common Root Rot	97.58	95.73	97.58	96.65
Fusarium Head Blight	98.08	97.14	98.07	97.60
Healthy	99.52	98.09	99.51	98.80
Leaf Blight	99.02	96.19	99.01	97.58
Mildew	97.20	99.52	97.19	98.34
Mite	98.11	98.57	98.11	98.34
Septoria	99.52	98.56	99.51	99.03
Smut	96.77	99.05	96.77	97.90
Stem fly	98.57	99.04	98.57	98.80
Tan spot	98.11	98.57	98.11	98.34
Yellow Rust	97.17	98.56	97.16	97.86

This discovery further demonstrates the model’s ability to accurately focus on samples at the interface of different classes, significantly reducing misclassification rates. The results underscore the superiority of our novel image augmentation method, along with the reverse learning and elimination of unnecessary features. Moreover, the additional incorporation of label information guaranteed strong feature consistency, facilitating the most precise feature responses. This enhances the model’s ability to accurately identify ambiguous samples at class boundaries and provides further evidence that reduces risk of misclassification.

## 4 Discussion

Automated classification of wheat diseases is essential in agriculture, significantly enhancing detection efficiency and accuracy, standardizing disease monitoring, and facilitating early identification of crop health problems. Examination of wheat diseases provides essential insights about the crop’s growing conditions. Variations in plant health—particularly the prevalence and severity of diseases such as rust, powdery mildew, and smut—serve as immediate indicators of the crop’s pathological condition. Regular monitoring of disease progression during cultivation enables precise assessment of crop health and productivity impacts, offering farmers critical insights to adjust planting strategies accordingly.

Moreover, automated wheat disease analysis techniques yield dependable and precise data, minimizing errors and subjectivity associated with manual assessment, hence improving the sensitivity and specificity of early detection. These technologies provide rapid diagnosis of wheat diseases, ensuring timely intervention before the condition worsens.

Thus, the creation and execution of a comprehensive automated wheat disease analysis system hold substantial practical importance in improving early disease detection rates, optimizing crop management strategies, and advancing the intelligence and efficiency of agricultural diagnostics.

This study presents an automated approach for identifying wheat leaf illnesses by regional feature purification contrastive learning. In the process of picture feature extraction, we employ unsupervised representation learning to optimize mutual information across varied data, while simultaneously applying mutual information maximization using image classification labels. This method implements improved localized constraints on self-supervised contrastive learning by including additional label information.

To enhance the semantic diversity of wheat leaf diseases, we amplify supervised contrastive learning to establish robust feature consistency and improve the model’s efficacy in out-ofdistribution detection. We devised the W-Paste methodology to generate affirmative cases. W-Paste simulates real-world situations concerning several types of wheat leaf diseases, hence enhancing the model’s robustness to input variations and its ability for out-of-distribution detection.

To thoroughly examine the semantic representations of wheat leaf diseases, we developed a feature purification encoder. In the early stages of training, we employ reverse learning to remove superfluous information from low-level features, isolate the most salient qualities by diminishing noise from intermediate features, and integrate high-level semantic features. This stratified approach significantly enhances the model’s effectiveness in identifying out-of-distribution instances. These distinctive strategies improve classification effectiveness and provide substantial support for the precise detection of wheat leaf diseases.

### 4.1 Limitations of study

Although our technology produces promising results, it nevertheless possesses certain limitations. While W-Paste augmentation has improved the model’s generalization to some extent, its efficacy may still be constrained when dealing with very complex illness samples. The current methodology heavily relies on the accuracy and availability of label information, which may, in certain cases, undermine the model’s robustness. Ultimately, despite the outstanding classification results obtained on public datasets, environmental variations and image quality in real-world applications may impact the model’s effectiveness. Therefore, future research should focus on evaluating the model’s applicability in real-world scenarios and consider including real-time data during training to enhance flexibility.

### 4.2 Future works

To improve the model’s generalization ability, it is recommended to develop a larger and more diversified dataset comprising wheat disease images from various geographical locations and climatic conditions. Furthermore, the application of semi-supervised learning approaches could leverage unlabelled data, enhancing the model’s effectiveness in scenarios with few labelled instances. Employing both labeled and unlabeled data would improve the feature extraction of wheat illnesses. Future research should focus on improving feature extraction techniques to more effectively differentiate between similar illnesses. Moreover, examining the use of self-attention processes in the feature extraction process could augment the model’s focus on critical features.

In the last stages of research, it is essential to assess the model’s effectiveness and robustness in real-world applications. Collaboration with farmers and agricultural experts to assess the model across many agricultural settings and get feedback for future improvements will be crucial. Ensuring the model’s ability to deliver accurate and reliable diagnoses in real-world situations will signify a significant progression toward facilitating its practical application. By exploring these paths, future research can significantly enhance the accuracy and relevance of automated wheat disease diagnosis, thereby building a solid foundation for the advancement of smart agriculture.

## 5 Conclusion

The detection of wheat diseases has consistently been a considerable problem in agricultural disease forecasting. Automated wheat disease classification is essential in contemporary agriculture, significantly enhancing detection efficiency and accuracy while promoting the standardization of disease monitoring, thereby enabling early diagnosis of crop health issues. The suggested method, utilizing regional feature purification contrastive learning, integrates unsupervised representation learning with label mutual information maximization to significantly improve feature extraction and classification efficacy for wheat leaf diseases. The implementation of the W-Paste approach enhances the model’s ability to manage input perturbations, hence augmenting its out-of-distribution detecting proficiency. The simultaneous development of a feature purification encoder enhances feature consistency, markedly improving classification accuracy.

Experimental results indicate that our method attains exceptional classification accuracy on public datasets, confirming its efficacy and resilience in intricate situations. This research presents a pragmatic approach for the automated identification of wheat diseases, establishing a basis for the advancement of intelligent agriculture. Ongoing enhancement of the model and methodologies is anticipated to yield greater progress in the early detection and accurate diagnosis of wheat illnesses in the future.

## Data Availability

The original contributions presented in the study are included in the article/supplementary material. Further inquiries can be directed to the corresponding author.
